# Barriers and facilitators towards recovery and health service utilization among Haredi Jews with mental illness

**DOI:** 10.1192/j.eurpsy.2023.751

**Published:** 2023-07-19

**Authors:** R. Whitley, E. Jarvis, M.-N. Le Blanc, E. Rohr

**Affiliations:** 1 Psychiatry, McGill University; 2 Sociology, University of Quebec in Montreal; 3 Medicine, McGill University, Montreal, Canada

## Abstract

**Introduction:**

Evidence suggests that minorities tend to under-utilize mental health services, and may face specific barriers and facilitators towards recovery. One community which remains particularly under-researched in the Western World are Haredi Jews — a diverse group of individuals characterized by a shared devotion to traditional Talmudic and Halakhah teachings and observances.

**Objectives:**

The overarching aim of this study is to document and analyze barriers and facilitators towards recovery and mental health service utilization among Haredi Jews with a history of mental distress. Specific objectives include: (i) eliciting and understanding participants’ mental health knowledge, beliefs, behaviours & attitudes; (ii) exploring their pathways and barriers to mental health care, especially examining the role of religiosity, religious community and rabbinical advice; and (iii) investigating their experience within the official mental health care system.

**Methods:**

To gain an in-depth understanding, we conducted a qualitative study. This involved semi-structured interviews with 24 participants who (i) identified as Haredi Jews; (ii) had used mental health services; and (iii) were 18+ years of age. It also included interviews with several key stakeholders, for example local Rabbis and other community leaders. Data was analyzed using thematic analysis techniques.

**Results:**

Participants typically had experienced mild to moderate mental distress, and tended to view mental health services in a positive light, mainly expressing satisfaction with services received. The analysis revealed three important facilitators and three important barriers to recovery. Facilitators comprised of (i) high levels of social support within the community, including specific well-being support groups; (ii) a positive relationship and connection with G-d, considered to provide guidance and support during troubled times; and (iii) the presence of many bridges and resources within the local Haredi community, including community-run health services, and Rabbis who encouraged mental health care utilization where appropriate. Barriers comprised of (i) stigma related to marriageability of self and offspring, inhibiting disclosure and mental health care use; (ii) acknowledged lack of awareness and knowledge about mental health, mental illness, treatments, and therapies; and (iii) generic health service issues, including long waitlists, limited availability and lack of appropriate therapists.

**Conclusions:**

Study participants tended to have positive views of psychiatric services, and utilized different health care and community-based resources to help foster recovery. However, ongoing issues of stigma and low levels of mental health literacy may inhibit mental health care use and recovery. This implies a need for religiously-informed and community-grounded mental health literacy campaigns among Haredi Jewish communities.

**Disclosure of Interest:**

None Declared

